# Observations on the Contagious Properties of the Varioloid Disease, or Modified Small-Pox

**Published:** 1825-06

**Authors:** Robert Venables

**Affiliations:** Physician to the Henley Dispensary, &c. &c. &c.


ART. III.—***Observations on the Contagious Properties of the Varioloid Disease, or Modified Small-Pox.*** By ROBERT VENABLES, M.D. Physician to the Henley Dispensary, &c. &c. &c.

THAT variola, in a modified form, occasionally appears in persons who have been subjected to vaccination, and have certainly undergone all the constitutional effects of regular cowpock, is a proposition too well established to be now questioned. But that the appearance of variola after regular vaccination is so frequent an occurrence as has been maintained by some, is a proposition, the truth of which is infinitely more doubtful. I have had extensive experience in small-pox, under every form and variety, and I can venture to assert, that, in the great majority of instances in which I have been informed that vaccination had been previously instituted, I could generally detect some irregularity or inconsistency in the history of the disease. When vaccination has been successfully practised, and variola subsequently attacks the same patient, I have almost invariably observed that the character of the latter is so far modified, as to be hardly recognisable from the appearances and progress of the variolous eruption. In the fourth volume of the Transactions of the King and Queen's College of Physicians in Ireland, a most respectable author, Dr. Joseph Clarke, makes the following observations upon this important subject:

"Much of the alarm which has arisen on this subject, and which still continues to agitate the public mind, appears to me to have been produced by want of due attention to a few words of Dr. Cullen's definition of variola:—'Papulæ phlegmonodeæ quæ *spatio octo dierum in suppurationem abeunt.*' Can it be deemed unreasonable to ask practitioners to keep these words constantly in mind, and to wait with patience till the eighth day after the eruption? If pus can then be found in the pustules, the existence of variola can no longer be doubted; but, until this period arrives, no prudent man should venture positively to decide. Let it be remembered that varicella is defined, by the same high authority,—'Pustulæ, variolæ similes, vix in suppurationem euntes, et post paucos dies desinentes.'" (P. 181.)

If these observations be received literally and in their fullest extent, it would lead to the inference that vaccination exerts no modifying influence upon the appearance and progress of the variolous eruption. Some facts, however, which have occurred under my own observation, seem to me decisive of this question. I shall state the following as most satisfactory.

When at Portsmouth in the year 1822, I was called upon by a soldier's wife, living in the Ordnance Barracks at this place, to visit her daughter, a girl about eleven or twelve years of age, who had been ill for two or three days. The child complained of cephalœa, was restless, feverish, and the eyes and nose portended some exanthematous affection. On examining minutely the state of the skin, there were a number of very small elevations, of a reddish appearance, and on the summit a degree of whiteness was just discernible. I then inquired if this girl had ever had small-pox, or been vaccinated? The mother told me that she had been vaccinated about seven or eight years before in Ceylon, and that the medical gentleman felt fully satisfied of the success of the operation. The febrile symptoms, though severe, were by no means dangerous, and the disease was going on progressively towards its termination. I myself considered it as a case of variola, modified by previous vaccination; and, on mentioning the circumstance to my friend, Dr. Jones, he expressed a wish to see it, and accordingly accompanied me to the barrack-room, where this patient was confined to bed. Dr. Jones remarked the anomalous character of the eruption, and pointedly directed my attention to the circumstance that its appearance by no means corresponded with that usually observed in small-pox at a similar stage of its progress.* He was inclined to consider the eruption as more allied, in its appear-

* At this period the disease was more advanced.

ance and general characters, to varicella.* This observation was certainly correct; but the mother having told me that the child had been near another who was labouring under variola, a short time before the febrile accession, and this accession having set in with some of the severe symptoms characteristic of the variolous eruptive fever, and the duration of the eruption being longer than is generally observable in varicella, I could not, under these circumstances, but regard the case as one of the varioloid disease.

Had the history of this disease terminated here, the relation of this case could have excited little or no interest, as the nature of the disease must still have remained, at least, problematical; though, for myself, I should have felt inclined to have bowed with deference to the opinion of my friend. The eruption went through its course without suppurating, merely hardening and turning into dry crusts, which fell off in the usual time, without leaving any pits. The nature of the disease, however, was soon afterwards placed beyond the possibility of doubt.

Those who are in the least degree acquainted with military regulations, know that the married men who live in barracks are, when it can possibly be done, quartered in rooms together. Hence it frequently happens that two, three, or four families, as the case may be, are quartered in the same room. This was the case here. There were several young children, some infants, in this room. Immediately on my suspecting the nature of this disease, I instituted vaccination with every child upon whom this operation had not been previously practised. With two the practice proved successful, and they escaped small-pox. In eight other instances variola made its appearance; and in three of those so severely, in the confluent form, that for a considerable period I anticipated an unfavourable result. I am happy, however, to add that my apprehensions proved groundless, as they all recovered, though much disfigured. In the other five instances the disease was severe, but not dangerous. In all the eight cases the eruption exhibited, clearly and unequivocally, the variolous character; being, in this feature, totally dissimilar from that whence they arose. The disease spread no further than this room, as I took the necessary precautions to prevent any intercourse; and it was thus speedily exterminated.

A question here, perhaps, arises,—is there any, and what, practical inference to be deduced from these facts? I answer, one, at least, of great importance, as to the propagation of the disease. Were we to pronounce such cases spurious, so far as

* These circumstances are merely mentioned to impress upon the reader the anomalous character of the eruption; and to prove, from the opinion of an accurate observer, that the eruption in no way resembled that of variola.

the contagious property of the disease is concerned, there can be little doubt that we should contribute to spread the disease through the unprotected part of a family, school, or district, and thus sacrifice our professional character. I am the more anxious to call the attention of the profession to the facts above stated, (and I could state others of a similar description, and equally satisfactory,) because I think some of Dr. Clarke's observations may be misunderstood, and hence may mislead. He says, "In several recent instances, variola and varicella have been confounded by practitioners of experience and ability. These errors must have arisen from opinions formed before the *duration* or *contents* of the pustules could be ascertained. The purport of these remarks is to guard my brethren against hasty and erroneous judgments, which unnecessarily disturb the peace of families, and tend to throw discredit on a practice eminently calculated to preserve human life, and to lessen human suffering." (P. 182.)

It may be questioned, however, if, from an over confidence in such a diagnosis, we should neglect proper precautions, and such as are necessary to prevent the propagation of a disease, against the contagion of which the constitution has not been previously fortified, it should unfortunately spread in a family, school, or district, we should not disturb the peace, and shake the confidence, much more than by adopting timely, though they might have been unnecessary, precautions. Had the circumstance which I have just related occurred in a respectable family or school, and unfortunately the disease had proved fatal to any one member, I fear the confidence of such would have been shaken, notwithstanding every effort, and a decidedly valuable and efficacious protection consigned to unmerited neglect. It is rather unfortunate that, in medical questions, the public reason not from actual facts, but from effects or consequences; and the neglect of vaccination would not be regarded as the source of fatality in the particular instances, but the mortality would be, however unjustly, set down to the account of the unprotecting influence* of vaccination experienced in any individual case. Dr. Clarke further states, "During the last few years I have seen many cases of spurious eruption, very like, in all symptoms, to severe small-pox, for the first eight, nine, or ten days from sickening. On the sixth day of the eruption, or at the latest the seventh, the pustules declined rapidly, the fever subsided, and on the eighth day not a vestige of pus was discoverable. Had these been cases of genuine variola, the patients, instead of amendment on the sixth day of

* I apply the expression "unprotecting influence" here, not to the severity or mortality of small-pox, but to its prevention.

eruption, would have remained in a state of increasing danger for many days, and would have experienced a slow recovery." (P. 182, 183.)

This description so well accords with the general progress of the modified small-pox, or varioloid disease, as described and named by Dr. Thomson, that there only remains one or two subjects of inquiry to identify them. Had the cases alluded to been vaccinated? Was there any communication with variolous patients; or was there any reason for presuming that these spurious cases arose from the contagion of variola? Did the spurious disease, in any one instance, give rise to genuine small-pox?

The case which I have related terminated in about eleven days;* and in this respect, and its general progress, bears a close analogy to Dr. Clarke's description; for about the eleventh or twelfth day there was scarcely a vestige of disease. There was no secondary fever; and this is a remarkable and characteristic feature of the modifying influence of vaccination upon the progress of variola. Yet this spurious disease produced small-pox in eight other individuals. I cannot imagine that these could have communicated the disease to each other, by one becoming first affected, and then infecting another, and thus communicating from one to another in succession; as they were all almost simultaneously affected, the whole eight sickening within three days.

Neither have I any reason to presume any other origin to the disease than what I have stated. I was particular in my inquiries as to whether they had any suspicious communication; but I discovered that the female parents of the children had not been out of the barrack for a considerable time, and that they had no acquaintance in the town, as the company to which they belonged had but lately arrived, and the children were too young to be trusted out by themselves. But with the first child it was different, as the company to which her father belonged had been at Portsmouth for a very considerable period, and the mother perfectly recollected the girl having been in a house in the town, where a child was lying either ill or but just convalescent from small-pox.

I by no means wish to inculcate that all the cases which present after vaccination are genuine small-pox, or that there are no spurious cases of this description devoid of contagious properties: on the contrary, I believe many of this nature have presented, and also that unfair advantage has been taken of the public alarm and credulity, from interested motives. I am anxious, however, to limit the observations of Dr. Clarke, and

---

* From the febrile accession, about the eighth day of the eruption.

to reduce his inferences within the precincts of safe and cautious principles of prophylaxis. If, upon the authority of Dr. Clarke, we were to regard every anomalous form of eruption as *spurious*, and hence incapable of infecting the healthy, but unprotected, with small-pox, I fear we should often have to regret our inattention to efficient preventive measures. Whenever, therefore, we have reason to suspect the nature of an eruptive febrile disease, however anomalous may be its character, we should adopt such precautions to prevent its spreading as the exigency of the circumstances may require.

I have lately, from some observations, reason to think that when the mild distinct small-pox attacks a person who has been previously constitutionally affected by cow-pock, that this form loses but little, if any, of its characteristic appearances; or, in other words, that it does not seem to be much modified* by the vaccine influence; but the confluent form is generally, as far as my observations enable me to determine, considerably modified, and the severity of the fever and of the eruption are both very much reduced under such circumstances.

In the varioloid disease, or modified small-pox, I have not observed those tendencies to severe local affections,—especially to inflammations which occasionally, indeed very frequently, appear in the unmodified confluent form. I have seen a great number of cases of the varioloid disease, and I cannot bring to my recollection a single instance of permanent organic derangement of the structure of the eye, nor of a similar or fatal derangement of the pulmonary, cerebral, or other visceral structures. Yet how often is loss of sight, and total destruction of the eye, the consequence of the unmodified confluent form of small-pox. How often have cerebral and pulmonary congestions, with all their train of concomitant symptoms, snatched the patient from the confident hopes of the physician and of the friends. How often has the patient fallen a victim to diseases which, from the remissions which had taken place, no human foresight could have anticipated, and no professional exertions have been able to avert. If vaccination afforded nothing more than a reasonably certain security against the direful sequelæ of so loathsome a disease, even this limited security would justly entitle it to our gratitude, and it should be hailed as one of the greatest blessings conferred by science upon suffering humanity.

*Henley; April* 12, 1825.

---

* This is merely thrown out as a subject worthy of future inquiry.

